# Using Functional Resonance Analysis Methodology to identify potential safety improvements in the process of administering medications by infusion in a veterinary hospital

**DOI:** 10.3389/fvets.2026.1781437

**Published:** 2026-03-30

**Authors:** Matthew McMillan

**Affiliations:** Faculty of Veterinary Science, LMU Small Animal Clinic, Ludwig-Maximilians University of Munich, Munich, Germany

**Keywords:** Functional Resonance Analysis Method, infusions, medication safety, patient safety, resilience engineering, veterinary

## Abstract

**Introduction:**

Medication error is a major cause of preventable harm in healthcare. Superficially, solutions to medication error appear simple, however medication administration often occurs during high-risk clinical scenarios, and under varied and challenging conditions which reduce intervention efficacy. Administering medication by infusion (AMI) is particularly complex. To develop feasible, effective and sustainable medication safety, requires an in-depth understanding of clinical conditions. Resilience engineering techniques can insight into process performance and variation and can identify how frontline staff can be better supported.

**Methods:**

A Functional Resonance Analysis Method (FRAM) based process evaluation was conducted in a multidisciplinary small-animal referral hospital. Information on work-as-imagined was obtained from institutional standard operating procedures (SOPs). Work-as-done was explored using an anonymous online staff survey with open-ended questions investigating process variation. Data were analysed using the four FRAM phases: identifying process functions, characterising performance variability, assessing functional resonance, and generating resilience-informed recommendations.

**Result:**

There were 45 responses to the survey included in analysis. The SOPs described a relatively linear process consisting of 7 phases whereas the FRAM model consisted of 45 interrelated and interdependent functions. Substantial discrepancies were identified between work-as-imagined and work-as-done. Performance variability and context dependent workarounds were common, particularly in relation to communication, dose calculators, infusion sheets, and double-checking, which were often impracticable under conditions of high workload, limited staffing, or clinical urgency. Interventions aimed at improving frontline abilities to anticipate, monitor and adjust to challenging conditions, and allowing the organisation to learn from outcomes were developed from the model.

**Discussion:**

FRAM demonstrated that AMI is a highly complex and variable process which is not adequately captured by existing SOPs. Performance variability reflected necessary adaptations to local conditions rather than individual non-compliance. Resilience-informed redesign of AMI processes may better support frontline staff and improve medication safety across diverse veterinary clinical contexts.

## 1 Introduction

Reducing medication error is considered a priority in human healthcare (1, 2). Data suggest 2%–4% of people receiving healthcare experience preventable medication-associated harm and that a quarter of this harm is either severe or life-threatening (2). Although data from veterinary healthcare are limited, existing studies suggest medication errors also harm veterinary patients (3, 4).

Improving medication safety is a complex challenge. Although the core phases of medication administration appear straightforward, i.e., prescribing, dispensing, preparing, checking, administering, documenting and communicating, and monitoring, they may encompass between 80–200 individual steps (5). Clinicians must perform these steps correctly in volatile, uncertain, complex and ambiguous environments, whilst managing multiple competing tasks and substantial resource constraints. Under such conditions, the medication-administration process is particularly prone to error. Indeed, increasing clinical pressure is associated with a greater risk of medication error, with emergency, intensive care, surgical care, and low-resource settings demonstrating especially high rates (2).

Intravenous administration, and in particular administering medications by infusion (AMI), adds further complexity and risk. These are associated with more challenging arithmetic, including unit conversions and dilution calculations, additional preparation steps, and the use of advanced technologies (6). In addition, intravenous medications are associated with high drug bioavailability, rapid increases in plasma concentration, an often-narrow therapeutic window, and is typically reserved for sicker and more critically ill patients reducing any margin for error (7). Despite the introduction of standardised protocols and smart infusion devices (SIDs) incorporating dose error reduction software programmed to calculate infusion rates within preset limits using a customizable drug library, medication errors during AMI still occur (6, 8).

Attempts to improve medication safety have traditionally centered on data from investigations on voluntarily reported safety incidents such as medication errors. Whilst these investigations provide useful insight into how things go wrong, they capture only a small part of a much larger complex system (5). Incident analysis typically compares what happened during an incident with how investigators believe the system operates or should operate, a concept termed *work-as-imagined* (5, 6). However, the complexity of healthcare means *work-as-imagined* often fails to reflect the reality of how work is actually performed on the clinic floor, the messy reality of *work-as-done* (5). To deliver care, staff must continually adjust their behaviour as they manage limited resources and competing tasks on multiple patients with a range of needs (5). For example, staff must dynamically balance between their work being thorough and being efficient, as it is not typically possible to optimise both. This is a principle termed as the efficiency-thoroughness trade-off (ETTO) (7–9). These adaptive adjustments can create unpredictable interactions between system functions and lead to disproportionate consequences, both positive and negative (6). As a result, interventions developed based solely on incident investigations tend to concentrate on individual compliance but may fail to integrate into local processes as they do not account for local conditions and challenges (10). This can greatly reduce their impact and lead to resources being wasted on ineffective, inefficient and unsustainable interventions.

Resilience engineering-based approaches consider the unpredictability of everyday work and how individuals adapt to, monitor, anticipate, and learn from changing conditions and how these factors lead to outcomes, both good and bad (6). The Functional Resonance Analysis Method (FRAM) is a resilience-engineering approach for examining processes and understanding how performance variability affects outcomes (11). The FRAM framework has been used in healthcare investigations and holds considerable potential for improving insight into how healthcare is actually performed and how staff can be better supported (12).

The primary objective of this study was to identify opportunities to improve the safety of AMI in a multidisciplinary specialist small-animal hospital through system redesign informed by resilience principles. To achieve this, FRAM was used to model *work-as-done*, identify the main sources of variation and deviation from *work-as-imagined*, and explore how system conditions shaped efficiency–thoroughness trade-offs in clinical practice.

Our hypothesis was that standard operating procedures (SOPs) defining how the process should be performed would not adequately reflect process performance under clinical conditions, that behavioural variability would be evident and shaped by local conditions, and that analysing this variability would reveal opportunities to better support adaptive performance.

## 2 Materials and methods

### 2.1 Study design

A FRAM-based process evaluation of AMI was conducted using data collected from SOPs and an anonymous online survey.

### 2.2 Setting

The investigation took place in a busy, multidisciplinary small-animal referral hospital in the United Kingdom. Despite the implementation of SOPs incorporating online drug calculators, double-checking protocols, individualised patient infusion sheets, smart infusion devices with internal drug libraries, pre-set safe dosing limits, and automated dose calculations, AMI-related medication errors and recurrent process problems continued to be reported.

### 2.3 Sampling and participants

Opportunistic sampling was used. All staff involved in AMI were invited to participate in the survey, including veterinary specialists, residents, interns, registered veterinary nurses, and student veterinary nurses. Staff worked primarily in one of four clinical areas: anaesthesia, wards, emergency and intensive care and pharmacy. Both permanent and locum staff were eligible to contribute.

### 2.4 Human ethics and data security

Only data from staff who gave informed consent for their responses to be used in this publication were included. Data collection and storage were anonymous and confidential. Personal identifiers and job-role information were not collected: only the individual’s role in AMI and clinical areas they worked in were recorded. Survey data were collected using a secure, academic, cloud-based platform (Qualtrics XM: Qualtrics LLC, Seattle, WA). Responses were downloaded immediately after survey closure and deleted from the platform. Data were stored in password-protected files on a single password-protected personal computer.

### 2.5 Investigator

The investigator had experience in relevant patient-safety theory, investigative methods, and analytical techniques but had received no formal training in FRAM.

### 2.6 Data sources

Information on *work-as-imagined* was obtained from hospital SOPs related to AMI. Information on *work-as-done* was gathered via an online survey consisting mainly of open-ended questions based on those recommended for use alongside FRAM (6). The survey is available as Supplementary file 1. The survey remained open for 4 weeks during which time two reminder emails were sent.

### 2.7 Analysis

The AMI process was analysed using the FRAM framework which comprises of four main phases (6).

#### 2.7.1 Phase 1: identifying functions

The essential functions (tasks, activities, steps) required to perform AMI in this hospital were identified and described. The aim was to capture the breadth and scope of the process rather than the depth and minutiae of each function. To support analysis, each function was described in terms of:

- What tasks and activities it involves
- Who performs it or needs to be present
- When it can be performed
- Where it can be performed
- Which resources are required to perform it safely and effectively
- How it is regulated
- Whether any conditions limit its performance or outcome

Using this information, functions were then defined using FRAM’s six aspects (6):

- Input: items transformed by the function (e.g., a treatment decision transformed into a medication order)
- Output: the result of the function (e.g., a prepared syringe or bag containing the intended dilution)
- Precondition: conditions that must exist before the function can occur (e.g., an accurate bodyweight for dose calculation)
- Resources: items required or used during the function (e.g., a fluid pump or syringe driver for medication infusion)
- Time: temporal constraints affecting the function (e.g., urgency influencing how long is available to complete a step)
- Control: mechanisms used to monitor, regulate, or verify the function (e.g., a double-check verifying a dose calculation).

#### 2.7.2 Phase 2: characterising variability

Any variability revealed in function performance was characterised, including internal variability (differences in how the function could be performed) and external variability (the influences arising from working conditions and resources which lead to internal variation). Variability was categorised as being related to timing (e.g., a function is performed too early, too late, or not at all) or precision (e.g., a function is partially performed or performed inadequately).

The conditions under which variation occurred, and the reasons for it, were also considered. Attention was paid to tensions, contradictions, compromises, and efficiency-thoroughness trade-offs made by staff to complete the process, and how these influenced function outcomes.

#### 2.7.3 Phase 3: assessing functional resonance

**Figure 1:**
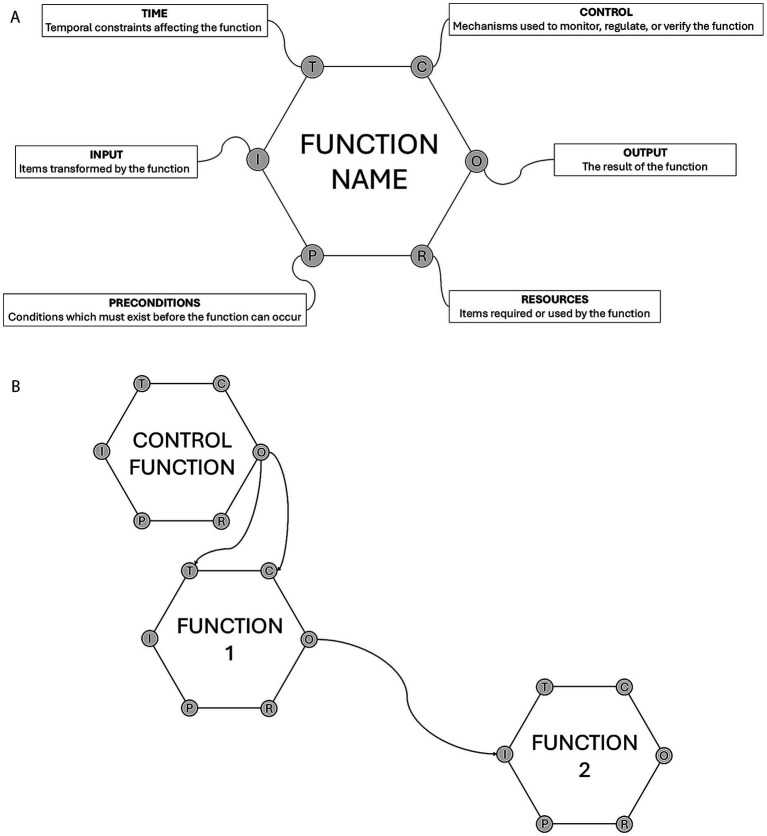
**(A)** How functions are depicted in the visual Functional Resonance Analysis Method model. Hexagons represent functions with each of its corners representing a function aspect: I, input; T, time; C, control; P, prerequisite; R, resource; and O, outcome. **(B)** How relationships and interdependencies are depicted in the visual Functional Resonance Analysis Method model. Lines between function aspects represent relevant interactions and arrows the direction of the relationship/influence. Adapted from (26); Reproduced under CC-BY-4.0.

How variability in one function could affect other functions and overall process outcomes was described, i.e., how variability could *resonate* throughout the system and influence subsequent functions and outcomes. Using the described functions, the AMI process was then mapped to develop a visual model of relationships and interdependencies. Each function was represented as a hexagon and each function aspect as a corner to this hexagon (Figure 1A). The relationship between each function was then depicted as a line running from the between the relevant aspects of the functions (Figure 1B).

#### 2.7.4 Phase 4: generating recommendations

Recommendations to optimise safe performance and reduce undesirable outcomes were then developed. These recommendations aimed to minimise unnecessary variability and to support staff where variation was necessary by promoting resilient behaviours which included improving the system’s ability to anticipate, monitor, respond and learn from changing conditions. Care was taken over designing interventions which preserved efficiency in settings and conditions where urgency was inherent to maintaining safety.

### 2.8 Evaluating the FRAM process

The time spent developing the FRAM model following data collection was recorded by documenting the start and finishing time for all sessions spent sorting and analysing data.

## 3 Results

### 3.1 Participants

Approximately 100 staff members were involved in AMI during the study period, split roughly 50:50 between veterinarians and RVNs/SVNs. Because of substantial changes in staffing and frequent use of locum personnel during this time, exact numbers could not be confirmed. The survey was accessed 47 times and completed by 45 participants. In terms of AMI settings, 24 participants were involved in wards, 19 were involved in ICU, 17 in anaesthesia and 5 in pharmacy. These numbers included 18 participants who were involved in more than one setting. Ordering medication was a role only reported by 17 participants, 15 of which who were also involved with all other phases. The remaining participants reported involvement with all phases other than prescribing.

### 3.2 Process complexity: work-as-imagined versus work-as-done

**Figure 2:**
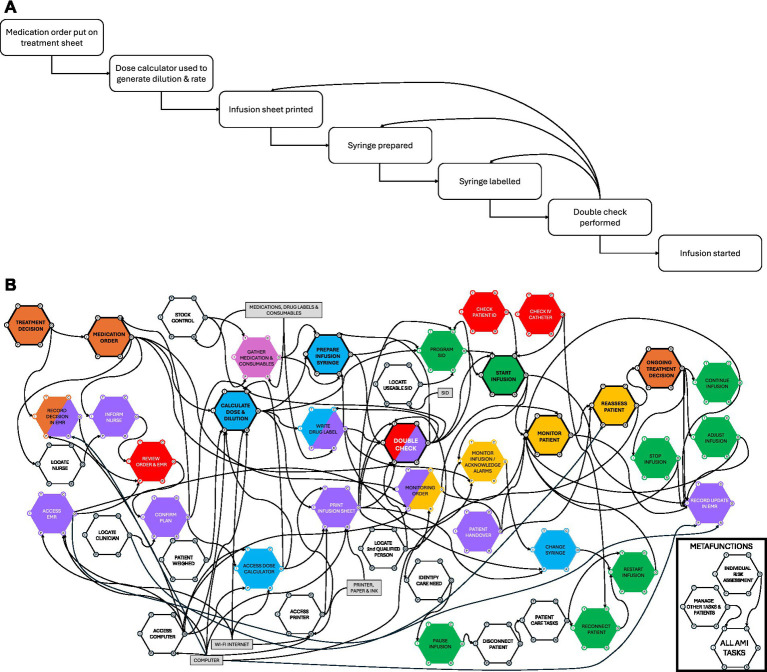
**(A)** A model of the designed seven task administering medication by infusion process as outlined in the standard operating procedures of a single multidisciplinary small animal hospital. **(B)** The developed Functional Resonance Analysis Method model of administering medications by infusion (AMI) in a multidisciplinary small-animal referral hospital. The model illustrates the key functions involved in AMI and their interconnections and suggests how variability in function performance can influence downstream functions and overall process outcome. Rectangles (grey) represent external resources. The colours are associated with the main process phases of prescribing (orange), dispensing (pink), preparing (blue), checking (red), administering (green), documenting and communicating (purple), monitoring (yellow), and background functions (white). Resources are depicted in grey boxes. Functions with thick black outlines, and bold, underlined font represent the core process functions which reflect those set out by the SOP. Please note that the numbers of the functions are there for identification purposes only and do not correspond to any sequential order, they correspond to the numbers used in Table 1 and in the Supplementary file 1.

Figure 2A presents the designed seven task AMI process as outlined in the hospital’s SOPs. Figure 2B presents the FRAM model describing the highly complex reality of work-as-done. This included 43 functions encompassing the main process phases of prescribing (orange), dispensing (pink), preparing (blue), checking (red), administering (green), documenting and communicating (purple), monitoring (yellow), and background functions which included two meta-functions which had global influence (white). These two diagrams highlight the significant differences between work-as-imagined and work-as-done. The FRAM model demonstrates substantially greater complexity and variability than was suggested by the SOPs and that different phases of the process could not neatly be separated, i.e., parts of different phases could occur concurrently and were spread throughout the process.

The dominant sources of variability identified across the key phases of administering medication by infusion and their consequences are reported in Table 1 (columns 1–4). Variation was frequently adaptive, allowing staff to respond to changing clinical conditions, resource constraints, and competing demands. The efficiency-thoroughness trades-offs made, and the variability they led to, often relied on staff experience, knowledge memory and recall, and therefore had the potential to contribute to ambiguity, error and patient harm. Of note AMI performed in anaesthesia and emergency care settings tended to favour efficiency over thoroughness, whereas in wards or intensive care settings thoroughness was favoured over efficiency. Favouring efficiency was sometimes felt to be safer as rapid processing allowed for more urgent and timely treatments.

**Table 1:** Summary of dominant sources of variation across administering medications by infusion process phases, the reasons for and consequences of these variations, and the resilience-based recommendations identified using the Functional Resonance Analysis Method in a single multidisciplinary small-animal referral hospital.

Phase [FRAM function]	Dominant variation(s) reported	Reported reason(s) for variation	Consequences [positive and negative]	Proposed intervention [resilience capability]
Prescribing [1–3, 31]	Mode of communication; written, verbal, both Channel of communication; electronic (multiple types), paper, in person, via intermediate Dose specificity; precise—vague Duration specificity; precise—vague Clinical reasoning; fully—partially—not communicated (assumed) Doses, rates, or dilutions; supported or unsupported by dose calculator	Assumed shared understanding/tacit knowledge; Clinical urgency; Easier communication; Experience; Interruptions/distractions; Task prioritisation; Workload/multitasking	[+] Rapid initiation of treatment; tailored therapy. [−] Ambiguity, misinterpretation, downstream uncertainty	Standardised prescribing templates with mandatory fields including documentation of clinical reasoning for medication, rationale for non-standard dosing/ dilutions, monitoring instructions, call orders & a range of doses, [anticipate, monitor]
Dispensing [12]	Medication sourced from different locations; local, other clinical area, main pharmacy Stock availability; available, unavailable, substituted Timing: immediate—delayed	Caseload; Clinical urgency; Dynamic stock levels; Physical layout; Stocking practice (balances expected local clinical demand & economic impact of wastage); Task prioritisation	[+] Economically viable & practicable, fits local usage patterns & limits wastage [−] Delays & workarounds	Develop a cultural habit of ensuring enough volume is prepared to last overnight, can be prompted in rounds & on dose calculator; visual stock status indicators & clear communication pathways for substitutions including at point-of-dispensing; indicator of priority included in medication order [anticipate, respond]
Preparing [11, 14, 16, 17, 37]	Calculation; dose calculator versus mental arithmetic Dose calculator used; updated, older versions Infusion sheets; printed or not Label type; pre-printed, hand-written Label contents; complete—partial—absent	Clinical urgency; Experience; Interruptions/distractions; IT access; Software limitations; Task prioritisation; Workload/multitasking	[+] Rapid preparation in emergencies [−] Increased calculation & labelling errors	Reliable access to correct dose calculators; Single sheet anaesthetic & emergency dose calculator sheets; Expanded dose calculator (may need to change software); Standardised labelling; Reduce out-of-hours preparation (adapt dose calculator to show when infusion will end/how long it will last, to trigger preparation of larger volumes or additional syringes, e.g., shows error message, include infusion check in rounds & handovers) [anticipate, respond]
Checking [8,21,23,24]	Double check scope; thorough—partial—none Double check depth; in-depth—superficial Double check performance; verbal sense-checking, artefact-based verification, full paper-based check	Clinical urgency; Local cultural norms; Staffing levels; Task prioritisation; Workload/multitasking	[+] Error interception [−] False reassurance when superficial, redundancy & inefficiency	Redesigned double-check process with defined components highlighted on infusion sheets; Integral automated double check process on dose calculator [monitor, anticipate, respond]
Administering [26, 27, 32–34, 38, 41, 42]	Programming; SID library, manual Reassessment; scheduled, *ad hoc* Active reassessment of infusion; present, absent Timing; immediately—delayed following preparation	Device limitations; Interruptions/distractions; Limited access to programming; Task prioritisation; Workload/multitasking	[+] Individualised therapy, adapts to requirements of clinical areas [−] Unintended dose changes, default continuation of infusions, failure to alter dose	Expand SID libraries, with medication s having different entries for wards, ICU & anaesthesia; Reassessment prompts; Visible stop criteria [monitor, respond]
Documenting & Communicating [3, 5, 7, 9, 17, 18, 21, 27, 35, 43]	Timing; before, during, or after administration Documentation; EMR, treatment sheet, anaesthetic record Handover content; thorough—nominal Handover presentation; structured—chaotic Handover documentation; notes taken, reliance on memory	Caseload; Interruptions/distractions; IT access; Task prioritisation; Workload/multitasking	[+] Flexibility under pressure [−] Reduced situational awareness, missed or poor documentation	Structured rounds with recording sheet and prompts to include infusions, monitoring and checks of volumes to be infused and current volumes prepared, Structured & harmonised documentation across IT platforms, Different sheets from different dose calculators, Regular team debriefings about infusion management, Simple one-click, online reporting of problems [monitor, learn]
Monitoring [27–30]	Monitoring order performance; verbal—written Monitoring order content; specific—vague (assumed) Monitoring performance; thorough—cursory Response guidance & call orders; explicit & action-oriented—discretionary escalation—absent Alarm response; immediate—delayed troubleshooting	Alarm fatigue; Assumed shared understanding/tacit knowledge; Clinical urgency; Communication quality; Staffing levels; Task prioritisation; Workload/multitasking	[+] Early detection of deterioration, maintains autonomy/clinical judgement [−] Delayed recognition, uncertainty of response	Monitoring goals & side effects included on infusion sheets, specific daily goals checklists, standardised rounds & handover to include specific infusions & monitoring sections [anticipate, monitor, respond]

[+] denotes positive consequences and [−] denotes negative consequences. *Handover* refers to any transfer of responsibility for a patient’s care between clinical teams or during a shift change within a team.

Importantly, several key control functions, i.e., using the online dose calculator, printing animal-specific infusion sheets, and performing double-checks, were often reported to be challenging or infeasible to complete as designed, particularly when the demand of a task or tasks exceeded available resources. Staff reported performing pragmatic, context-dependent risk assessments to decide whether and how to carry out these control measures. These situational compromises were more common during anaesthesia, emergencies, or out-of-hours work.

Staff also identified difficulties locating essential resources (Figure 1B, grey), especially smart infusion devices, computers, printers, and available colleagues as inhibiting the process. Communication challenges were reported regarding the clinical reasoning for an infusion being started, ongoing need for an infusion, monitoring requirements, and adjustments to infusion rates. These issues reflected weaknesses in the current design of the AMI process and background system functions.

### 3.3 Recommendations and interventions

The main recommendation was that SOPs be redesigned to acknowledge the weaknesses in control measures and account for efficiency-thoroughness trade-offs which were necessary to maintain safety in different settings, namely anaesthesia and emergencies.

Proposed interventions focussed on shaping and supporting variability toward safe and effective performance rather than eliminating it. They involved reducing reliance on memory and tacit knowledge by externalising critical information into tools, artefacts, and workflows and supporting clinicians’ ability to anticipate, monitor, respond to, and learn from changing conditions without constraining adaptive performance (Table 1, column 5). These included developing different tools for different areas to support thoroughness when efficiency was critical.

## 4 Conclusion

Overall, the findings indicated that the SOPs contained multiple shortcomings and did not fully reflect the full range of clinical conditions encountered by staff or how AMI could be practicably performed in some clinical areas and situations. The use of FRAM helped identify these shortcomings and develop targeted interventions to support anticipation, monitoring response and learning. The application of efficiency-thoroughness Trade-off principles allowed interventions to be designed which maintained efficiency during time-critical situations.

### 4.1 Evaluation of the FRAM process

The process was time consuming, taking approximately 40 h to complete. Although the generated model clearly demonstrates the complexity of the process and the oversimplified nature of current SOPs, its accuracy has not been validated. Whilst the concept was intuitive and variation and workarounds were simple to identify, characterising variation and the interactions and interdependencies between functions was more challenging.

## 5 Discussion

The AMI process in this hospital could be mapped by FRAM and highlighted both performance variability and the discrepancies between work-as-imagined and work-as-done. Existing SOPs presented an over-simplified, idealised, rigid and linear version of AMI which failed to reflect the reality of local working conditions and frontline process performance. In contrast, FRAM revealed AMI as a highly complex, non-linear process characterised by multiple interacting and interdependent functions. The analysis identified widespread variation in performance, the adaptive strategies used by staff to manage this variability, and the downstream consequences of these adaptations for system performance and outcomes.

Indeed, variation was identified across many functions, including those involved in communication, record keeping, labelling syringes, using dose calculators, and importantly performing control measures such as double-checks. Staff commonly encountered resource limitations particularly staffing levels, available expertise, and deficits in equipment and technology. Time constraints and intense demand regularly required staff to split their attention, prioritise tasks, multitask and make rapid risk assessments. The findings of this study, reflect the ETTO principle, with staff often making dynamic trade-offs between being thorough and being efficient (7, 8). These trade-offs varied considerably by setting with thoroughness being favoured in ward and ICU settings, and efficiency in emergency and anaesthesia settings. Where efficiency was prioritised, this was often associated with staff being faced with urgent situations, which were reported as being more commonplace during anaesthesia and emergency case management. In these situations, efficiency in task performance led to patients starting medications in a timelier fashion which enabled their condition to be more rapidly stabilised. This finding challenges the assumption that safety is dependent only on thoroughness (8). Indeed, it appears clinical safety can be rooted in both thoroughness and efficiency, and too much of either can lead to failure (7). Safety can be manifested through thoroughness dominating and ensuring all key tasks being performed, and through efficiency dominating and avoiding the process being performed too slowly which leads to treatment delays (7). Such findings have also been identified in the process of medication prescribing in primary human healthcare (8).

Cumbersome and inefficient workflows including fragmented information flows, multiple communication channels, and SOPs which did not account for all clinical scenarios requiring AMI, frequently led staff to need to use workarounds to efficiently and effectively complete tasks. These workarounds were informed by experience-based, pragmatic, context-dependent risk assessments and subsequent situational trade-offs, e.g., prioritising one aspect of a task over another. Different clinical areas, i.e., intensive care, anaesthesia and wards, described distinct conditions and challenges and tended to perform functions and workarounds in slightly different ways. All these variations had the potential to influence staff behaviours, task performance and outcome in both positive and negative ways.

Several functions designed to control performance and ensure accuracy, e.g., using online dose calculators, printing infusion sheets for each infusion, and conducting full double-checking procedures, were described as impracticable under many common clinical conditions, e.g., out-of-hours periods, low staffing levels, situations requiring multiple infusions in a short time, or emergencies. A non-standardised patient assessment and handover process appeared to add unnecessary variation and might increase risk through failing to provide control of associated functions.

The recommendations developed from this evaluation focused on developing interventions to reduce unnecessary variation and adjusting the AMI-related SOPs to better support staff across a wider range of realistic working conditions. Interventions aimed to strengthen staff ability to anticipate, monitor, and respond to changing circumstances and to enhance the organisation’s capacity to learn from successful frontline adaptations.

The present analysis suggests that no single solution or SOP can ensure the safety of AMI given the complexity of the process and the range of clinical contexts in which it occurs. An SOP which functions adequately in a ward setting may be overly prescriptive or impractical in anaesthesia or emergency contexts, where rapid decisions and actions are a prerequisite for success. This does not imply that existing SOPs are wrong, but rather that they are incomplete and require adaptation and augmentation to accommodate specific system challenges. More importantly our analysis suggests that control measures such as the dose calculator, drug library, double checking process, handover and communication procedures, need to be adapted to better reflect staff needs and local conditions.

Numerous FRAM-based investigations in human healthcare support these observations (12). Studies have applied FRAM to medication administration (13–16), referral and diagnostic pathways (17), sepsis management (18–20), medical device use (21), handovers and transitions of care (22, 23), discharge processes (24, 25), follow-up care (26), safety incidents (27), and the implementation of safety interventions (28). Collectively, these studies have provided insight into healthcare system functioning, including the identification of critical process functions, the inherently variable nature of performance, mismatches between demand and available resources, conflicting rules and recommendations, and the shifting nature of risk within a process (12). For example, Kaya and Hocaoglu (29) demonstrated that variability in function performance can lead to both successful and unsuccessful outcomes. Schutijser and colleagues (28) found that time pressure, interruptions and resource mismatches contributed to omissions or superficial performance of medication double-checks. Similarly, Clay-Williams and colleagues (30) identified process functions that conflicted with local implementation requirements for clinical practice guidelines. Each these studies demonstrated the need for staff to perform workarounds to perform specific functions within processes in clinical conditions (12).

### 5.1 Limitations

This study has several limitations. First, the investigation was performed at a single centre. Although the point of such an investigation is to establish work-as-done patterns at a local level this may limit the generalisability of the findings. Variation in SOPs, staffing levels, workload, workflows and organisational culture across settings may constrain the transferability of the present FRAM model and reduce the potential effectiveness of the proposed interventions in different hospitals. Nevertheless, many of the challenges identified, e.g., resource constraints, time pressures, and the need for adaptive workarounds, are common across clinical environments and may therefore have broader relevance.

Second, FRAM is a conceptually demanding, interpretive method which can be challenging and time consuming to perform comprehensively (6, 11). The method is a non-prescriptive framework which requires significant analytical judgement to perform properly (11). The flexibility which allows FRAM to be applied across diverse contexts also introduces subjectivity much like other qualitative methods (11). Differentiating between different aspects of a function, e.g., whether something is a prerequisite or a resource, can seem somewhat semantic and may not particularly help with developing the model or potential solutions. Although established FRAM guidance was followed, these are not aimed specifically at healthcare. This required significant interpretation and reference to previous FRAM studies performed in healthcare (11–27). Furthermore, the investigator had no formal FRAM training, which may have influenced how well functions and variability were identified and characterised. Although the concepts of FRAM are accessible, can be used by non-experts, and are supported by publicly available guidance, its effective application requires more than following online instructions. The investigator therefore had to undertake a considerable amount of self-directed study of FRAM principles, published examples, and resilience engineering concepts to conduct the analysis and develop the model. The most obvious problem with the method seen in this study is that the final model is extremely complex, messy and difficult to follow. Whilst the model visually demonstrates the complexity and interdependencies between functions, it does not help understanding beyond this. Indeed, the functional tables set out in the supplementary materials perhaps offer a far more understandable way to evaluate and redesign the process and develop interventions.

Third, the analysis relied primarily on information gathered from an anonymous online survey to develop a model of work-as-done. Although this enabled broad participation, it did not allow in-depth follow-up, so may have failed to identify some of the nuances of frontline practice. Using semi-structured interviews might develop a more in-depth view of the process but this method is considerably more time consuming and challenging to perform. Social desirability bias may also lead staff to alter their responses to make it seem as though their behaviour fits in with organisational expectations. Direct observation may have captured even more subtle behaviours and variations, particularly those involving tacit knowledge or embedded workarounds which are not easy to articulate. Observation however is very time consuming as to capture the degree of variation experienced in the AMI process, many hours of observations were likely to be required. These alternative methods were not considered feasible for the current study.

Finally, although the recommendations generated were based on an analysis of work-as-done, they do not guarantee improved safety in the AMI process. Their impact must still be evaluated in practice to determine whether they meaningfully support staff, reduce unnecessary variability, and improve outcomes. Although FRAM provides a valuable foundation for understanding system performance, it represents only the first step in an iterative process of testing, refinement, and organisational learning.

## 6 Conclusion

Resilience engineering-based investigation methods such as FRAM offer considerable potential to inform practice and improve safety (12). Resilience engineering conceptualises variability as an inherent property of complex systems which enables work to be completed and outcomes to be achieved under variable and challenging conditions (31). This is in stark contrast to traditional safety investigations which commonly treat performance variability as an individual-level deviation from idealised care which requires control (31). Resilience engineering acknowledges that staff must make continual adjustments and trade-offs, e.g., between efficiency and thoroughness, to perform their duties when faced with a lack of alignment between work demands and available resources (9). Furthermore, resilience engineering accepts that not everything can be successfully proceduralised and protocolised in a complex adaptive system, and that deviation at some point will become necessary to maintain functionality (6). From this perspective, poor performance and unwanted outcomes are not understood simply as individual failings but as signals that the system is not adequately supporting frontline work (5, 6, 31). By focusing on *work-as-done*, resilience engineering identifies where variability and trade-offs are necessary for successful performance under local conditions. Therefore, solutions can be developed to support staff navigate these challenging conditions safely (11, 31).

Although FRAM is challenging and time-consuming to perform, it revealed important realities about the AMI process and exposed weaknesses within the hospital’s SOPs and control measures. The analysis demonstrated that SOPs cannot reasonably be expected to capture the full complexity of AMI, given the wide variation in clinical contexts in which the process occurs. Consequently, compliance with SOPs should not be relied upon as the sole mechanism for ensuring safety. By using FRAM, the gaps between *work-as-imagined* and *work-as-done* can be identified and used as a basis for developing recommendations to support safer and more efficient performance. Whether these recommendations will lead to measurable improvements remains to be determined. Nonetheless, FRAM offers a potentially valuable approach to safety improvement which can complement incident investigations by providing a richer understanding of the local realities of clinical work.

## Data Availability

The original contributions presented in the study are included in the article/Supplementary material, further inquiries can be directed to the corresponding author.
