# Supplementary material for: Using Functional Resonance Analysis Methodology to identify potential safety improvements in the process of administering medications by infusion in a veterinary hospital
Source: Front Vet Sci. 2026 Mar 30;13:1781437. doi: 10.3389/fvets.2026.1781437 (PMC13072484; doi:10.3389/fvets.2026.1781437)
Supplement: Supplementary file 1 [file supplementary_file_1.pdf]

Supplementary materials 1: Survey

Questions asked in the staff survey on the local process of and their role in administering medication by infusion

1. Which of the following tasks in the process of administering infusions of medications to patients are you involved in? (please tick all that apply)

   - Ordering a medication to be infused
   - Dispensing a medication to be infused (e.g., getting the drug from pharmacy or a stock location)
   - Preparing a medication to be infused (e.g., preparing a dilution and syringe)
   - Administering a medication by infusion (e.g., programming a syringe driver or fluid pump, connecting it to a patient)
   - Checking an infusion calculation
   - Documenting or communicating an infusion
   - Monitoring and reassessment of a patient receiving an infusion of medication (e.g., watching for effectiveness and side effects of medications, deciding when to change a dose rate or stop an infusion)

2. In which area do you undertake this role (multiple may be chosen)?

   - Wards
   - Anaesthesia
   - ICU
   - Pharmacy

3. To complete your role(s) in administering medication s or fluids by infusion what tasks do you perform? (Please, give as much detail as you can, e.g., first I get information about the patient from X, then I get information about the medication from Y. Then I gather everything I need from Z, before… etc)

4. Thinking about each of the tasks you are involved in when administering medication s or fluids by infusion: How do the tasks you perform in the process of setting up and administering a drug by infusion vary case-by-case? (e.g., sometimes, I have to…)

5. What things (e.g., equipment, information etc.) do you need to perform your role(s) in setting up and administering medications by infusion?

6. Does the availability or function of these things vary? (e.g., do you always have everything you need, and does it always work the way you need it to?)

7. What do you do if something unexpected happens whilst you are performing your role(s) in setting up and administering a medication by infusion (e.g., should there be an interruption or a problem)?

8. What do you do if there is time pressure on setting up and administering an infusion?

9. What do you do if information is missing, or if you cannot get hold of certain people?

10. Do you ever need to customize, adjust, or adapt any of the tasks involved with setting up and administering medications by infusion to ensure it can be completed effectively and efficiently? If so, what do you do and why?

11. Is there something that you must often tolerate or get used to when performing your role in setting up and administering a drug by infusion?

12. How often do you have to change the way you perform your role(s) in setting up and administering a medication by infusion for example to be more efficient?

    Likert: Every time, Usually, Frequently, Sometimes, Occasionally, Rarely, Never

    Roughly how many times out of ten, do you think you have to change the way you perform this task?

13. Have you encountered any problems when performing any of the tasks involved in your role in administering medication s or fluids by infusion? Please outline what these problems were and how you solved or worked around them?

14. Do you feel the control measures in place to ensure the process is performed correctly are practicable to your role, e.g., are they available, efficient and practical to use? Consider the dose calculator, double checking, handover communication, clinical record keeping etc

15. Please describe in your experience why setting up and administering a medication by infusion generally succeeds?

16. Please describe in your experience how things can go wrong with setting up and administering a medication by infusion?

17. Thinking about each role you have in the process of setting up and administering a medication by infusion, and the current standard operating procedures we have in place, how would you improve the way this process can be performed?

18. If you were going to improve or change anything about how we set up and administer medication by infusion in this hospital what would you change? Please feel free to make multiple recommendations

	Function	Phase	Input	Time	Control	Preconditions	Resources	Output	Variation
1	Treatment decision	Prescribing	Clinical exam, history, patient update / <br>reassessment, diagnostic results	Based on clinical urgency <br>†				Medication & dose decision	Different clinicians may elect to treat problems and manage symptoms in different ways
2	Medication order	Prescribing	Medication & dose decision	†	Plan confirmed			Complete medication order	May be written or verbal or both
3	Record decision in EMR	Prescribing, Documenting & Communicating	Medication order	†	Background functions: network monitoring & maintenance, software updates	Computer Access, Wi-Fi functioning, software up to date	Computer, Wi-Fi internet	Updated EMR	May be recorded just on treatment sheet or on the EMR as well <br>Written record may be filled out by veterinarian or nurse
4	Locate Nurse	Background function	N/A	†	Background function: Staff numbers & shift management			Verbal communication possible	Clinician may have to set up infusion themselves
5	Access EMR	Documenting & Communicating	N/A	†		Computer Access, Wi-Fi functioning, software up to date	Computer, Wi-Fi internet	Logged into EMR	Access may not be available
6	Locate clinician	Background function	N/A	†	Staff experience & individual risk assessment	Clinician not in consults or performing a procedure		Verbal communication possible	May need to refer to an alternative clinician or decide that a

									communication is not necessary
7	Inform Nurse	Documenting & Communicating	Complete medication order	†		Nurse located		Medication order verbally communicated	Order may just be written on treatment sheet
8	Review medication order & EMR	Checking	Nurse informed	†		Access to EMR<br><br>Medication order recorded on treatment sheet and EMR<br><br>Full details of medication order recorded		Medication order reviewed	May not get performed<br><br>May be specific or vague<br><br>Clinical reasoning may not be imparted
9	Confirm plan	Documenting & Communicating	Clinician located	†	Staff experience & individual risk assessment			Confirmed medication plan	May not be performed<br><br>Can range from a closed-loop, formal full check of medication requirements through to a cursory confirmation
10	Stock control	Background function	N/A	†				Available medications and consumables	Stock levels in different areas change dynamically and are often depleted locally require collection from central stores

11	Calculate dose & dilution	Preparing	Medication order	Based on clinical urgency<br><br>†	Dose calculator<br><br>Double check<br><br>Plan confirmation<br><br>Staff experience & individual risk assessment	Computer Access<br><br>Dose calculator access<br><br>Wi-Fi functioning Medication and dose listed in "catalogue"<br><br>Accurate current weight	Computer, Wi-Fi internet, pocket calculator	Calculated dilution and infusion rate	Can be calculated using the central dose calculator, other dose calculation software, using a pocket calculator, or by mental arithmetic
12	Gather medication & consumables	Dispensing	Dose calculation	Local stock levels<br><br>Based on clinical urgency<br><br>†		Stock control	Medications and consumables	Required medications and consumables gathered for dilution and assembly	Where medications and consumables are gathered from varies, e.g., central pharmacy, ICU and anaesthesia drug stores, local consumable stores, main consumable stores
13	Patient weighed	Background function	N/A	†				Accurate current patient weight	May be gained by admitting clinician or by nursing staff or support service<br><br>May not be up to date if patient hospitalised

14	Access Dose Calculator	Preparing	Confirmed plan	Based on clinical urgency<br><br>†	Background function: Software checks and updates<br><br>Staff experience & individual risk assessment	Login details	Computer, Wi-Fi internet	Ability to perform "authorised" dose calculations	Clinical urgency ranges enormously
15	Access Computer	Background function	N/A	Based on clinical urgency<br><br>†	Background function: Hardware maintenance and troubleshooting	Login details	Computer	Access to EMR and dose calculator<br><br>Printing of infusion sheet	Although many computers available local access, especially point-of-care, can still be limited
16	Prepare infusion syringe	Preparing	Calculated dilution and infusion rate	Based on clinical urgency<br><br>†	Dose calculator<br><br>Double check<br><br>Clinical experience		Medications, consumables, prepared syringe label	Prepared syringe<br><br>(Labelled syringe of diluted medication ready to be infused)	Due to the size range and differing dosing requirements of patients, dilutions and volumes can vary as to their suitability
17	Write Drug label	Preparing, Documenting & Communicating	Calculated dilution	Based on clinical urgency<br><br>†	Dose calculator		Syringe label, permanent pen	Prepared syringe label	Drug label may be medication specific or generic<br><br>Information required on each varies
18	Print infusion sheet	Documenting & Communicating	Dose calculated	Based on clinical urgency	Double check	Access to printer	Computer, printer, paper and ink	Prepared infusion sheet	May not be performed in anaesthesia, may be performed after

				Access to printer<br><br>†	Staff experience & individual risk assessment	Access to dose calculator			infusion has started
19	Access printer	Background function		†	Background function: Printer maintenance and troubleshooting		Computer, printer, paper and ink, Wi-fi internet	Ability to print infusion sheet	Many areas do not have direct access to a local printer Central printers may be out of service or some distance form point-of-care
20	Locate useable SID	Background function	N/A	Based on clinical urgency<br><br>Overall hospital caseload<br><br>†			SID, power cable	Ready to use SID	Although SIDs are allotted to each area demand can be very variable meaning they often need to be borrowed<br><br>May require taking an SID from another patient
21	Double check	Checking, Documenting & Communicating	Medication order<br><br>Prepared infusion sheet & syringe	Locating 2nd qualified person<br><br>†	Staff experience & individual risk assessment	Available 2nd qualified person located and available<br><br>Infusion sheet printed<br><br>All preceding functions completed as per SOP	Prepared infusion syringe or composite parts (medication plus diluent in separate syringes)<br><br>Printed infusion sheet	Verified dilution, dose and programmed SID	What is verified during the double check process can differ, e.g., a mixture of the infusion sheet, with the medication order, the original medication vial, the prepared

							Medication order<br><br>Programmed SID		fluid bag or syringe and label, the programmed smart infusion device<br><br>Staff just sign to say a check has been performed not what that check consisted of
22	Locate 2nd qualified person	Background function		Based on clinical urgency<br><br>†	Background function: Management of staff numbers<br><br>Staffing rotas			Suitably qualified person available to perform double check	The availability of staff varies massively with workload and clinical priorities
23	Check patient ID	Checking	Background function	Based on clinical urgency and prior knowledge/ relationship with patient<br><br>†	Staff knowledge of & relationship with patient & individual risk assessment	Animal has a fully completed ID collar placed		Confirmed animal ID	In some situations, the animal may not have an ID collar placed, e.g., emergency admit, or has neck bandage /wounds<br><br>May not be performed if staff know the animal
24	Check IV catheter	Checking	Background function	Based on clinical urgency<br><br>†	Staff experience & individual risk assessment		Saline flush syringe	Patent ready-to-use IV catheter	May not be checked if recently placed and used

25	Program SID	Administering		Based on clinical urgency<br><br>†	In built medication library with dose calculation and predefined thresholds<br><br>Infusion sheet	Background functions: Up to date medication library<br><br>Correct drug dose and dilution chosen	SID, prepared infusion syringe	Prepared infusion syringe in a programmed SID	Drug dose and/or dilution may not be in the drug library requiring bypass of the dose calculator and thresholds
26	Start infusion	Administering	Prepared infusion syringe in a programmed SID	Based on clinical urgency<br><br>†	Background functions: SID functions e.g., high pressure alarm	Patent ready-to-use IV catheter	SID, prepared infusion syringe, sufficient battery or wired power source	Animal receives medication by infusion	The time the infusion gets started varies depending on a priority decision and perceived clinical urgency
27	Monitoring order	Monitoring, Documenting & Communicating	Medication order	Based on clinical urgency<br><br>†	Assessment of the experience of staff assigned to monitor<br><br>Staff experience & individual risk assessment			Specific monitoring regarding infusion is set out	This may or may not be done and even if done may not be recorded on EMR or treatment sheet
28	Monitor patient	Monitoring	Monitoring order	Based on clinical urgency<br><br>†	Background functions:<br><br>Treatment sheets<br><br>Twice daily patient checks	Monitoring orders on EMR	Computer, Wi-fi internet	Animal receives specific monitoring regarding infusion	No specific instructions about monitoring patient, thresholds or likely side effects may be given (assumed knowledge)
29	Monitor infusion & acknowledge alarms	Monitoring	Animal receiving receives medication by infusion	†	Background functions: SID functions e.g.,				Monitoring may occur as part of twice daily assessments, general

					high pressure alarm				observations triggered by a change on patient status by nursing staff, or specifically associated with orders
30	Reassess patient	Monitoring	Change in status identified<br><br>Background function: Twice daily patient assessment performed	†		Clinician located		Change in status identified	May or not get triggered between daily checks depending on
31	Ongoing treatment decision	Prescribing	Patient ongoing needs identified	†				Patient ongoing needs identified Patient infusion plan made	Can be delayed which can lead to animals not having infusions adjusted to their needs
32	Continue infusion	Administering	Patient infusion plan made	†				Continued infusion	Default often to continue and this may not be necessary<br><br>Often continued as a default
33	Stop infusion	Administering	Patient infusion plan made	†				Stopped infusion	Often stopped quite some time after it was truly needed<br><br>May not be clear when the

									infusion should be stopped
34	Adjust infusion	Administering	Patient infusion plan made	†				Adjusted infusion	Often not adjusted regularly enough<br><br>May not be clear under which circumstances the infusion should be changed
35	Record update in EMR	Documenting & Communicating	Patient infusion plan made: Stop, continue or adjust	†		Access to EMR (& computer)	Computer, Wi-fi internet	Updated infusion plan on EMR	May or may not be performed<br><br>May have very scant details
36	Identify care need	Background function	Patient needs reassessed	†				Patient care need planned	May happen once or twice a day or very regularly
37	Change syringe	Preparing	Syringe empty / running low	†			Infusion sheet<br><br>Written drug label containing dilution information	New syringe prepared	Can take some time leaving animals without infusion, often not done in time with pauses for care needs
38	Pause infusion	Administering	Care need identified	†				Paused infusion	Can be paused for a very variable amount of time

39	Disconnect patient	Background function	Paused infusion for care need	†			Bungs for infusion and IV catheter	Disconnected patient	
40	Patient care tasks	Background function	Patient care need planned	†				Patient care needs performed	Hugely variable needs, frequencies and durations
41	Reconnect patient	Administering	Patient care needs performed	†			Full infusion set up still available	Animal reconnected to infusion	Sometimes SIDs are needed for other patients
42	Restart infusion	Administering	Animal reconnected to infusion	†				Infusion restarted	
43	Patient Handover & Rounds	Documenting & Communicating	Handover in care or shift change	†				Critical clinical information communicated between care teams & shift changes	Handovers can be incomplete, disorganised and poorly structured<br><br>Notes might not be taken
44	Manage other tasks & patients †	Metafunction	N/A	Caseload, workload	Hugely variable	Hugely variable	Hugely variable	Hugely variable which has knock effects on all other functions including itself	Hugely variable based on caseload, staffing, disease/injury complexity, patient needs etc
45	Individual Risk Assessment	Background function	Whenever a decision about how to proceed is required	†	Staff knowledge, experience, confidence, local culture			Controls the decision on how to proceed	Hugely variable based on task prioritisation, risk aversion, situation awareness, experience,

									confidence, local culture especially expectations of work efficiency

Supplementary materials 2: Table defining functions in terms of their six Functional Resonance Analysis Method Aspects: Input, Time, Control, Resources, Prerequisites and Outputs. The table is based upon a Functional Resonance Analysis Method performed in a single multidisciplinary small animal referral hospital. A metafunction is a function that effects all or most other functions within the process without really being part of that function, all functions identified as being affected by the metafunction are marked with †.

	Function	What is done?	Who does it involve?	When is it done?	Where is it done?	Which resources does it need?	How is it regulated?	Whether conditions limit its performance?
1	Treatment decision	A decision is made on an animal's treatment	Clinician	Variable time following initial assessment and then after any change in state or reassessment	Any area in hospital		Twice daily rounds trigger treatment decisions	Caseload, workload and clinical urgency affect timing and duration of function
2	Medication order	A infused medication is entered onto the animal's treatment sheet or verbally communicated to nursing staff	Clinician +/- nurse	Variable time after treatment decision is made	Typically wards but can occur anywhere in hospital, e.g., medications can be ordered from the clinician's office	Computer, Wi-Fi internet, functional software	Nurse will enter medication order onto system if not done by clinician	The amount of multitasking being performed can affect thoroughness and completeness of performance
3	Record decision in EMR	A note of the treatment decision is made in the animals EMR	Clinician or nurse	Some time after treatment decision made	Anywhere	Computer, Wi-Fi internet, functional software		Wider workload can mean this task is not performed
4	Locate Nurse	Clinician locates nurse	Clinician and nurse	Sometime after treatment decision and either during verbal medication order or after written order	Anywhere but typically wards	Telephone, messenger services		Multiple places a nurse can be in the hospital and may not be able to answer phone
5	Access EMR	The EMR record of the animal is access by staffed	Clinicians and nurses	Multiple time points. Whenever new data need to be entered, or something needs to be checked	Anywhere in hospital or off-site via remote access	Computer, Wi-Fi internet, functional software		Can take a while to log into patient's EMR

6	Locate clinician	Nurse locates clinician	Clinicians and nurses	Whenever there is a change in patient status or something needs to be clarified or reported	Anywhere but typically wards	Telephone, email, messenger services		Multiple places a clinician can be in the hospital and may not be able to answer phone
7	Inform Nurse	Information on medication order communicated to nurse	Nurses, clinicians, interns, residents	Whenever a change in treatment requirements is made	Anywhere but typically wards	Telephone, messenger services	Backed up on treatment sheet	Time constraints may mean this step is missed and the infusion is only ordered on the treatment sheet
8	Review medication order & EMR	Nurse checks the medication order	Nurse	When medication order received	Anywhere but tends to be wards or pharmacy	Computer, Wi-Fi internet, functional software		Time constraints and other tasks with higher priority may mean this is performed in a cursory manner or not performed at all
9	Confirm plan	Nurses confirm the medication order	Nurses, clinicians, interns, residents	As soon as possible after medication order received	Anywhere, often by phone or messenger service	Telephone, messenger services		Busy clinicians may not be receptive to this confirmation which may lead nurses to avoid this step
10	Stock control	Clinical area kept stocked with medications and consumables	Nurses, animal care assistants, pharmacist, management	Ongoing task	All clinical areas and stock rooms	Inventory, medications and consumables		Can be affected by national medication or consumable shortages, staff levels, workload and caseload

11	Dose & Dilution Calculation	Required dose rate, dilution and volume calculated	Nurses, clinicians, interns, residents	As soon as possible after medication order received (and confirmed)	Pharmacy, ward, ICU or anaesthesia areas	Computer, Wi-Fi internet, functional software	Double check, dose calculator Pocket calculator	
12	Gather medication & consumables	All the required medications and materials such as syringes and infusion lines are collected	Nurses, interns, residents	After dose and dilution calculations have been made	Pharmacy, ward, ICU or anaesthesia areas, stock room	Medications and consumables		Affected by stock management and local usage, may require going to various locations to gather the required materials
13	Patient weighed	Patient's bodyweight is measured and entered into the animal's EMR	Nurses, clinicians, interns, residents, animal care assistant	When admitted then at least once daily	Various sites	Electronic scales		When pushed for time often not performed or performed and not recorded, maybe only entered onto EMR or treatment sheet
14	Access dose calculator	Staff log onto a cloud-based dose calculator to automatically calculate dose rates and dilutions	Nurses, clinicians, interns, residents	Once medication order received or after confirmation	Pharmacy, ward, ICU or anaesthesia areas	Computer, Wi-Fi internet, functional software		Computer access is limited and smart phone compatibility poor
15	Access Computer	Staff log onto a local computer with Wi-Fi internet access	Nurses, clinicians, interns, residents	Whenever access to EMR, treatment sheet or dose calculator needed	Any area of hospital			Computer access is limited and smart phone compatibility poor
16	Prepare infusion syringe	A syringe of medication at a set dilution is prepared and labelled	Nurses, clinicians, interns, residents	After dilution calculation performed	Pharmacy, ward, ICU or anaesthesia areas	Medications, needles, syringes, infusion lines, completed, syringe label	Dose calculator<br><br>Double check<br><br>Infusion sheet	Often a lot of noise and distraction<br><br>May be one of multiple task an individual is

								entrusted with at any point in time
17	Write syringe label	A label outlining the medication, dilution, date and patient details is prepared	Nurses, clinicians, interns, residents	After syringe has been prepared	Pharmacy, ward, ICU or anaesthesia areas	Drug label, permanent pen	Dose calculator<br><br>Double check<br><br>Infusion sheet	May need to be administered in time critical situations, may just get a medication identification label rather than complete dilution information
18	Print infusion sheet	An individualised infusion sheet outlining doses, rates and dilutions is printed for reference purposes	Nurses, clinicians, interns, residents	After dose has been calculated using dose calculator	Pharmacy, ward, ICU or anaesthesia areas	Printer, Computer, Wi-Fi Internet	Nurse asks for infusion sheet if none printed<br><br>Noted at rounds	Often infusions needed to be started in a time critical fashion, e.g., under anaesthesia or in emergencies. This step is time consuming and a redundancy if the drug library and dose calculation function of SIDs are used
19	Access printer	A functioning printer (e.g., with ink, paper, no current errors) must be located	Nurses, clinicians, interns, residents	Before infusion sheet is printed	Reception, scheduling station, offices	Computer, Wi-fi Internet, Printer, Paper, Ink		Printers only available in a few areas. Some of these are not in clinical areas. Often paper jams or the ink or paper has run out.
20	Locate useable SID	A functioning SID is located and secured for the	Nurses, clinicians, interns, residents	Before programming the	ICU, anaesthesia, wards, imaging	SID	Specific SIDs labelled for	There is often a shortage of SIDs in specific areas meaning staff

		animal requiring the infusion		SID and starting the infusion	suit, operating rooms		specific clinical areas	must try to find an SID in other clinical areas
21	Double check	A second suitably qualified person checks the medication order, infusion sheet, dose, rate, dilution and label of the intended infusion syringe	Nurses, clinicians, interns, residents	During or after preparing the infusion syringe	ICU, wards (rarely anaesthesia)	Drug order, Infusion sheet, prepared infusion syringe		The double check can be performed in several different ways depending on the time the person performing the double check can spend on the assessment<br><br>Distractions and other clinical duties often take priority leading to only a cursory check being performed
22	Locate 2nd qualified person	The person setting up the infusion finds a second person to verify the infusion will be administered as prescribed	Nurses, clinicians, interns, residents	Before a double check can be performed	Any clinical area		Staffing levels maintained by hospital management	Finding the second person can be challenging, finding someone who is not involved in performing another task or in managing an animal is even harder<br><br>Out-of-hours and during anaesthesia or emergencies this can be especially challenging

23	Check patient ID	The ID of the animal is checked	Nurses, clinicians, interns, residents	Before the patient is connected to the infusion or the infusion is started	ICU, wards, Anaesthesia, Operating rooms	ID collar, Computer with access to treatment sheet and EMR	Nurses confirm collar is on animal during first care interaction	If busy animals may be put in a kennel by a clinician without a collar<br><br>Day patients may arrive to anaesthesia without a collar<br><br>Animals admitted in an emergency needing urgent stabilisation may have treatment prioritised over ensuring patient identifiable
24	Check IV catheter	The catheter bandage is removed and the catheter is flushed with saline to confirm patency and absence of pain on injection	Nurses, clinicians, interns, residents, animal care assistants	Before the patient is connected to the infusion	ICU, wards, Anaesthesia, Operating rooms	Saline flush syringe	Written up for 4 times daily flushing	This can be difficult to achieve if animal in dorsal recumbency and covered in drapes in an operating room, may also have been recently flushed
25	Program SID	The SID is programmed with information on the medication, dilution and dose. Rates are automatically calculated	Nurses, clinicians, interns, residents	Before the infusion is started	ICU, wards, Anaesthesia, Operating rooms	SID with power cable or adequate battery power, prepared syringe with infusion line, patent IV catheter	Should be checked during twice daily assessments, when patient status changes, and when disconnected/reconnected	In busy periods animals may just be reconnected without checking the rate, dilution etc

26	Start infusion	The infusion is started	Nurses, clinicians, interns, residents	After the SID has been programmed and infusion attached to animal's IV catheter	ICU, wards, Anaesthesia, Operating rooms	SID with power cable or adequate battery power, prepared syringe with infusion line, patent IV catheter	SID alarm functions, e.g., high pressure, failure to press start	Can forget to press start, this is generally identified after about a minute as the SID alarms
27	Monitoring order	A list of parameters to be specifically monitored associated with he effects and side effects of the medication are entered onto the animal's treatment sheet or communicated with the nurse managing the case	Clinicians, residents, interns	Any time during the process, but should be before the infusion is started	ICU, wards, Anaesthesia, Operating rooms	Computer, Wi-fi Internet, access to treatment sheet, telephone, messenger service	Should be considered and communicated during rounds if not already performed	Information may get lost due to shift patterns and handovers. Weekend and out-of-hours work especially problematic as fewer staff and higher workload.<br><br>In anaesthesia this is often not written down as the pace too high and the tasks too numerous for this to be achievable most of the time. May not be performed elsewhere as these factors are either assumed, or the clinician has limited time available to communicate a comprehensive monitoring plan.
28	Monitor patient	The effects and side effects of the medication are	Clinicians, residents, interns, nurses, student	Continuous or continual throughout	ICU, wards, anaesthesia, operating rooms	Computer, Wi-fi Internet, access to treatment sheet	Twice daily patient assessments,	The amount of attention an animal receives is

		monitored alongside general changes in the animal's status	nurses, animal care assistants	infusion and animal's hospital stay			treatment sheet schedules	dependent on its perceived clinical needs, workload, staffing levels, how well information on the patient has been communicated and the animal's behaviour amongst other factors
29	Monitor infusion & acknowledge alarms	The volume left in the syringe is checked to ensure a new syringe does not need to be prepared<br><br>When alarms sound the SID, syringe, infusion line and catheter are checked	Clinicians, residents, interns, nurses, student nurses, animal care assistants	Continuous or continual throughout infusion and animal's hospital stay and whenever alarm sounds	ICU, wards, anaesthesia, operating rooms	Functioning SID	Twice daily patient assessments, plus 2 hourly infusion checks	May not be checked during assessments as assumed has been checked multiple times. Infusion checks often not performed fully every two hours as intended, typically it is checked whether the infusion is running, less regularly the rate is compared to the infusion sheet, rarely are the contents of the syringe checked at these times
30	Reassess patient	The animal is reassessed to establish current status and ongoing needs	Clinician, resident, intern	Before handovers/rounds<br><br>Whenever triggered by a	ICU, wards, anaesthesia, operating rooms		Twice daily rounds and handovers<br><br>Triggers on treatment sheet or	Full reassessment may not be performed depending on

				change in animal status			anaesthetic record	workload and staffing levels
31	Ongoing treatment decision	A decision is made about how best to proceed with the infusion	Clinician, resident, intern	Whenever a reassessment has been made	ICU, wards, anaesthesia, operating rooms	Computer, Wi-Fi Internet	Twice daily rounds and handovers<br><br>Triggers on treatment sheet or anaesthetic record	When the hospital is busy, default often to continue infusions unless specific plan laid out
32	Continue infusion	The infusion is continued at its previous rate	Clinician, resident, intern, nurse	After ongoing treatment decision	ICU, wards, anaesthesia, operating rooms	SID with power cable or adequate battery power, prepared syringe with infusion line, patent IV catheter	Monitoring orders	
33	Stop infusion	The infusion is stopped as it is no longer needed	Clinician, resident, intern, nurse	After ongoing treatment decision	ICU, wards, anaesthesia, operating rooms	Caps for IV and infusion lines	Monitoring orders	
34	Adjust infusion	The infusion is adjusted to meet the animal's needs	Clinician, resident, intern, nurse	After ongoing treatment decision	ICU, wards, anaesthesia, operating rooms	SID with power cable or adequate battery power, prepared syringe with infusion line, patent IV catheter	Monitoring orders	
35	Record update in EMR	The reassessment is recorded in the EMR	Clinician, resident, intern, nurse	After ongoing treatment decision	ICU, wards, anaesthesia, operating rooms	Computer, Wi-Fi Internet		When busy or juggling many tasks and patients, clinicians may neglect this task
36	Identify care need	Staff notice that the animal needs to be taken out of its kennel for some reason	Nurse, student nurse, animal care assistant, intern, resident, clinician	Whenever there is something due to happen to the animal or when there is a change in status or the	ICU, Wards		Treatment sheet, patient reassessments, walk by observations	Depends on the workload, number of animals being manged and the staffing levels

				animal needs to have its basic needs addressed				
37	Change syringe	Empty syringe gets replaced by a newly prepared full syringe	Nurse, student nurse, intern, resident, clinician	Whenever the syringe appears to be nearly empty or whenever the SID alarms about low volume or being empty	ICU, Wards, Anaesthesia, Operating rooms	Medications, needles, syringes, infusion lines	SID alarms	At critical times alarms may be silenced and SIDs put into standby whilst other tasks with higher priority are performed
38	Pause infusion	Infusion paused to allow disconnection or syringe change	Nurse, student nurse, animal care assistant, intern, resident, clinician	Whenever the patient needs disconnecting or syringe needs changing	ICU, Wards, Anaesthesia, Operating rooms			
39	Disconnect patient	Animal disconnected from infusion	Nurse, student nurse, animal care assistant, intern, resident, clinician	Whenever a clinical requirement or patient care need	ICU, Wards, Anaesthesia, Operating rooms	Caps for IV and infusion lines		
40	Patient care tasks	Patient care tasks are performed (clinical or basic needs)	Nurse, student nurse, animal care assistant, intern, resident, clinician	Whenever required	ICU, Wards, Anaesthesia, Operating rooms	Variable		
41	Reconnect patient	The animal is reconnected to the infusion	Nurse, student nurse, animal care assistant, intern, resident, clinician	Following completion of a patient care task	ICU, Wards, Anaesthesia, Operating rooms	SID with power cable or adequate battery power, prepared syringe with infusion line, patent IV catheter		
42	Restart infusion	The infusion is restarted	Nurse, student nurse, animal care assistant, intern, resident, clinician	After the animal is reconnected following a patient care task	ICU, Wards, Anaesthesia, Operating rooms	SID with power cable or adequate battery power, prepared syringe with infusion line, patent IV catheter		

43	Patient handovers & rounds	Whenever there is a shift change or handover in care information about a patient and their management including infusions is communicated	All staff	Whenever required, end of every shift	ICU, Wards, Anaesthesia, Operating rooms		Staff can ask questions if they are unsure	Staff maybe dealing with other patients or even involved with procedures when handovers or rounds are due to take place. They maybe shortened because of other duties
44	Manage other tasks & patients †	Performance of all clinical and non-clinical tasks and patients within the hospital	All staff	Continuous	All hospital areas	All hospital infrastructure, equipment, medications and consumables	Managerial and organisation oversight	Significant staffing and other resource limitations and a fluctuating caseload with variable patient complexity and needs
45	Individual Risk Assessment	A risk assessment is made on how to proceed	All staff	Whenever a decision needs to be made about ongoing care or actions/inactions	All hospital areas			Dependent on workload, staffing, resources, information available, but also on the condition of the individual and how they are effected by the environment and conditions

Supplementary materials 3: Table defining each function of Administering Medicine by Infusion in terms of What is done, Who it involves, When it is done, Which resources are required, How it is regulated and Whether specific conditions limit its performance. The table is based upon the Functional Resonance Analysis Method performed in a single multidisciplinary small animal referral hospital.
